# Effect of screw insertion depth into fractured vertebrae in the treatment of thoracolumbar fractures

**DOI:** 10.1186/s13018-024-05026-x

**Published:** 2024-10-16

**Authors:** Jinghuai Wang, Dong Ren, Lindan Geng, Yufeng Chen, Shuangquan Yao, Pengcheng Wang

**Affiliations:** 1 Orthopedic Trauma Service Center, Major Laboratory Orthopedic Biomechanics in Hebei Province, Third Hospital of Hebei Medical University, Shijiazhuang, China; 2 Department of Orthopedics, Affiliated Hospital of Hebei Engineering University, Handan, China

**Keywords:** Thoracolumbar fracture, Insertion depth, Intermediate screws, Endplate reduction

## Abstract

**Purpose:**

The study’s objective was to assess the effect of the screw insertion depth into fractured vertebrae in treating thoracolumbar fractures.

**Materials and methods:**

This was a retrospective analysis of 92 patients with thoracolumbar fractures from December 2018 to February 2020. Patients had AO type A2, A3 thoracolumbar fractures. The patients were divided into two groups according to the screw insertion depth. The vertebral wedge angle (VWA), Cobb angle (CA), anterior vertebral body height (AVBH), middle vertebral body height (MVBH), visual analog scale (VAS) score, and Oswestry Disability Index (ODI) were compared preoperatively and at one week and 12 months postoperatively. The correlation between Vertebral height loss and potential risk factors, such as sex, age, BMD and BMI was evaluated.

**Results:**

Compared with the preoperative data, the postoperative clinical and radiographic findings were significantly different in both groups, But no significant difference between the two groups at 1 week. At 1 year postoperatively, there was a significant difference in the CA (*p* < 0.0001), VWA (*p* = 0.047), AVBH (*p* < 0.0001), MVBH (*p* < 0.0001), VAS score (*p* < 0.0001), and ODI (*p* < 0.0001) between the two groups, Except for age, bone density and other influencing factors the long screw group had better treatment results than the short screw group.

**Conclusion:**

A longer screw provides greater grip on the fractured vertebral body and stronger support to the vertebral plate. The optimal screw placement depth exceeds 60% of the vertebral body length on the lateral view.

## Introduction

Thoracolumbar fractures are the most common type of spinal fracture, accounting for approximately 90% of spinal fractures. T11 to L2 are within the thoracolumbar spine [1], a segment with high mobility and stress concentration that is prone to burst fractures [2, 3].

Restoring the alignment and stability of the spine to prevent kyphosis are the primary goals of treatment for thoracolumbar fractures [4]. While there are a variety of surgical methods to treat thoracolumbar fractures, the best treatment method remains controversial [5]. Early surgical approaches include anterior fixation, posterior fixation or combined anterior and posterior fixation [6–8]. The clinical results of anterior surgery are established, but the anterior approach is difficult, causes extensive bleeding, and carries high surgical risk [9]. Posterior surgery is more acceptable because of its ease and relatively low surgical risk. Compared to traditional long-segment fixation, short-segment fixation allows for less surgical invasion, a shorter operation, less bleeding and less soft tissue damage [10, 11]. Biomechanical and clinical studies have shown that short-segment pedicle screw fixation is effective in maintaining the reduction of lumbar fractures [12–15]. As a result, posterior short-segment fixation has been widely used for thoracolumbar fractures. However, the failure of vertebral implants used in short-segment fixation and problems such as kyphosis and pain have been reported [16, 17]. Thus, an increasing number of scholars have begun to study the placement of screws at the fracture level to overcome the shortcomings of the “quadrilateral” in short-segment fixation, which is insufficient to achieve stability of the injured segment of the spine, optimize the stress distribution within the internal fixation, increase the holding force of the fractured segment of the pedicle, and further reduce the occurrence of retroflexion deformity. However, the question of how deeply the screws should be placed at the fracture level remains.

In this study, we focused on the depth of screw insertion into the fractured vertebra with the aim of assessing the effect of different screw insertion depths in the treatment of thoracolumbar fractures.

## Materials and methods

### Patient population

**Fig. 1:**
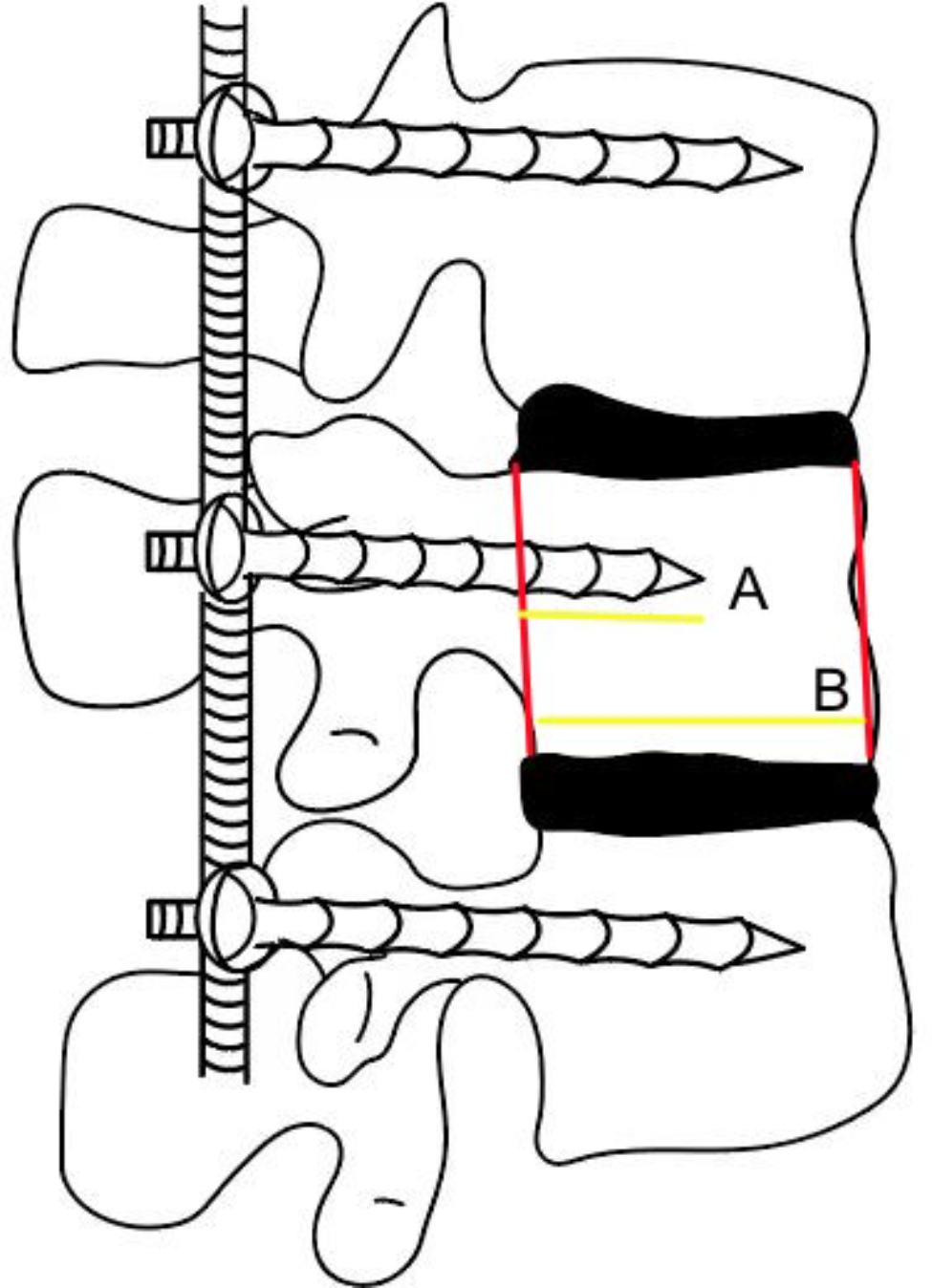
Screw insertion depth measurement method: A/B, where **A** is the screw insertion length, and **B** is the vertebral body length

**Fig. 2:**
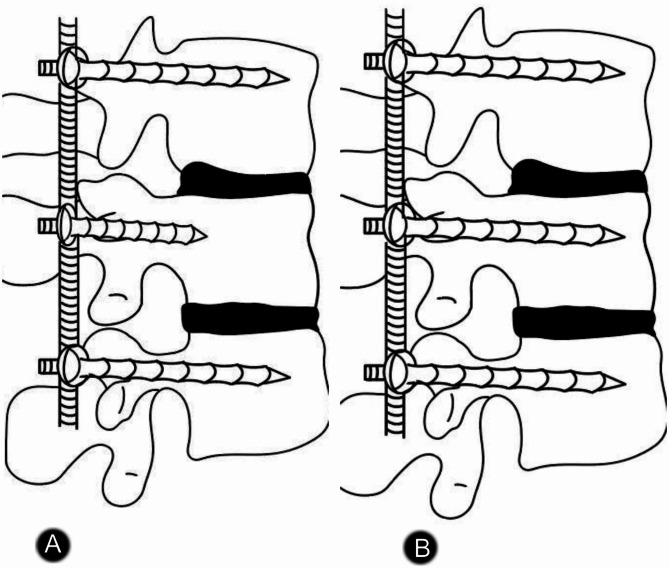
**A**, The short screw (SS) group, In this group, on the lateral view, the insertion depth did not surpass the posterior edge of the vertebral body and was no greater than 50% of the vertebral body length. **B**, The long screw (LS) group, In this group, on the lateral view, the insertion depth was more than 60% but not greater than 80% of the vertebral body length

Ninety-two patients with thoracolumbar fractures were admitted to our department between December 2018 and February 2020 and received treatment with posterior short-segment fixation. The research protocol (2018206314) was examined by the Hebei Medical University Ethics Committee before being approved. All patients provided their informed consent for this research. The inclusion criteria were as follows: (1) single-segment thoracolumbar fractures of type A3, A4, or B1; (2) treatment within 3 weeks of injury; (3) aged 18–60 years; (4) no evidence of nerve root damage; and (5) ability to complete at least one year of follow-up. The exclusion criteria were as follows: (1) a serious underlying illness; (2) many segmental injuries; (3) osteoporosis; and (4) a thoracolumbar fracture with an arch fracture. Depending on how far the pedicle screws were inserted into the vertebral body, the patients were split into two groups. The screw insertion depth was determined by dividing the length of the screw in the vertebral body by the anterior-posterior diameter of the vertebral body. The long screw (LS) group consisted of 53 patients. In this group, on the lateral view, the insertion depth was more than 60% but not greater than 80% of the vertebral body length [18]. The short screw (SS) group consisted of 39 patients. In this group, on the lateral view, the insertion depth did not surpass the posterior edge of the vertebral body and was no greater than 50% of the vertebral body length (Figs. 1, 2; Table 1) [18].

**Table 1:** Summary of demographic and clinical data distribution in both groups

Parameter	LS group	SS group	*P*
Number of patients	54	39	
Age, mean ± SD	43.52 ± 13.51	48.67 ± 12.37	0.081‡
Sex, male/female	35/19	23/16	0.566^†^
Body mass index (kg/m ^2^)	23.39 ± 1.48	23.24 ± 1.16	0.285^‡^
Bone mineral density, mean ± SD	0.70 ± 1.29	0.38 ± 1.20	0.218^†^
Fracture level			0.713^†^
T11	10	6	
T12	4	3	
L1	20	18	
L2	13	7	
L3	4	5	
AO-Magerl classification			0.515^†^
A2	30	19	
A3	24	20	
Fracture mechanism			0.829^†^
Traffic accident	29	19	
Fall from height	20	15	
Others	5	5	

Values are shown as the number, mean, and standard deviation. (%). LS group, long screw group; SS group, short screw group

*p < 0.05 was considered statistically significant. † Chi-square test. ‡ Wilcoxon test

Assessment of the patient’s age, sex, cause of trauma, degree and type of fracture.

### Surgical technique

**Fig. 3:**
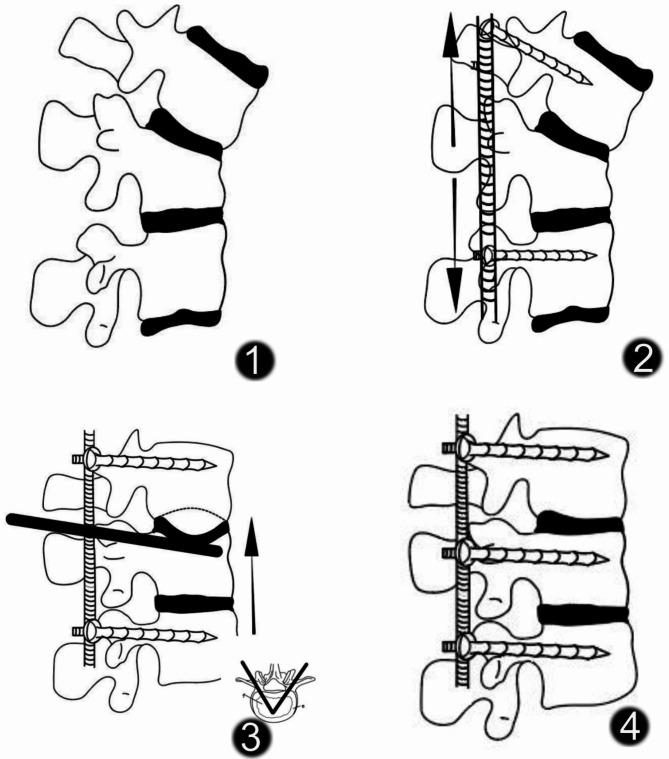
**1,** fractured vertebrae. **2,** the fractured vertebrae were repositioned. **3,** intermediate screws were inserted into the fractured vertebrae. **4,** Simultaneous repositioning of the collapsed endplate via both sides of the pedicle using a special tool

The patient was positioned in a prone posture with a pad to support the pelvis and midbody after general anesthesia was successfully established. Using a posterior median approach, the paravertebral muscles on either side of the spinal eminence were dissected to expose the pedicle screw insertion points. Screws were first placed above and below the fractured vertebrae, followed by installation of the connecting rods; then, the fractured vertebrae were repositioned by propping up the ipsilateral screws with dilators. Simultaneous repositioning of the collapsed endplate via both sides of the pedicle using a special tool. After satisfactory repositioning, intermediate screws were inserted into the fractured vertebrae, and the connecting rods and screws were locked (Fig. 3).

All procedures are performed by the same operator, who has a senior title, and the instruments used in the procedures were provided by the same manufacturer.

### Postoperative management

Standard antibiotics were administered to all patients for 48 h after surgery to prevent infection. Patients were instructed to vigorously move their legs while taking anticoagulant medication during bed rest to prevent deep vein thrombosis in the lower limbs. After the drainage tube was removed on the third postoperative day, the patients were advised to continue wearing a thoracolumbar brace to ensure proper mobility.

### Clinical and radiographic review

**Fig. 4:**
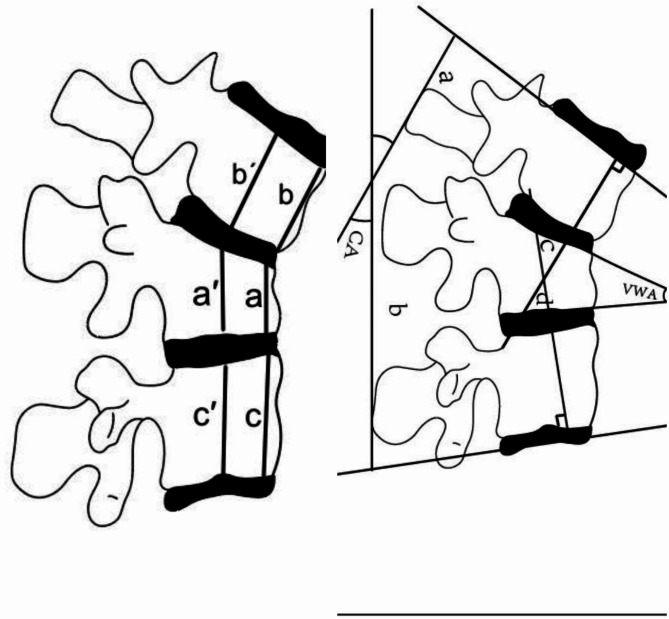
Cobb angle (CA) measurement method: the angle formed by lines **a** and **b**. Vertebral wedge angle (VWA) measurement method: the angle formed by lines **c** and **d**. Anterior vertebral body height (AVBH) measurement method: AVBH = 2 a/(b + c)×100%. Middle vertebral body height (MVBH) measurement method: MVBH = 2 a’/(b’+c’)×100%

Clinical and radiographic results from the 1-week and 1-year postoperative follow-ups were evaluated in this study. The clinical data evaluated were the visual analog scale (VAS) score and the Oswestry Disability Index (ODI). The radio-graphic data evaluated were the Cobb angle (CA), vertebral wedge angle (VWA), anterior vertebral body height (AVBH), and middle vertebral body height (MVBH) (Fig. 4). The vertical line that is produced by the lower end plate of the lower vertebral body and the higher end-plate of the upper vertebral body forms the Cobb angle. The angle created between the upper and lower endplates of the fractured vertebral body is known as the VWA. The AVBH is created by the ratio of the anterior height of the fractured vertebral body to the average height of the anterior portions of the upper and lower adjacent vertebral bodies. The MVBH is the average height of the middle of the upper and lower vertebral bodies adjacent to the fractured vertebral body to the height of the middle of the fractured vertebral body (Fig. 4) [9].

### Statistical analysis

In this research, statistical analysis was performed to compare the preoperative and postoperative follow-up results between the two groups and identify correlations between the preoperative and postoperative follow-up data. The data were processed using SPSS 24.0 (IBM, Armonk, NY, USA). A statistically significant difference was indicated by probability values lower than 0.05. For properly distributed continuous variables, the independent-samples t test was employed, while nonparametric tests were utilized for variables that did not follow a normal distribution. Data are presented as the mean and standard deviation. For categorical data, Fisher’s exact test and Pearson’s chi-square test were used. Logistic regression analyses were performed to exclude confounding factors such as age, sex, and BMD and to correct for covariates.

## Results

In terms of age, sex, cause of trauma, and degree of fracture, the two groups were comparable (*P* > 0.05). The LS group consisted of 54 patients in total, including 19 females and 35 males. The average body mass index (BMI) was 23.39 ± 1.48 kg/m2. Among these cases, the etiology was related to a fall in 20 cases, a traffic accident in 29 cases, and another injury in 6 cases; vertebral fracture occurred at T11 in 10 cases, T12 in 7 cases, L1 in 20 cases, L2 in 13 cases, and L3 in 4 cases. The SS group consisted of 39 patients, including 16 females and 23 males, with a mean age of 48.67 ± 12.37 years. The average body mass index (BMI) was 23.24 ± 1.16 kg/m^2^. Among these cases, the etiology was related to a fall in 15 cases, a traffic accident in 19 cases, and another injury in 5 cases; vertebral fracture occurred at T11 in 6 cases, T12 in 3 cases, L1 in 18 cases, L2 in 7 cases, and L3 in 5 cases. At baseline and one week after surgery, there were no statistically significant differences between the LS and SS groups in terms of sex, age, BMI, mode of injury, or fracture location.

The CA was maintained at 4.36 ± 0.62° in the LS group compared to 8.78°±4.16° in the SS group at the 1-year postoperative follow-up; however, there was a statistically significant difference (*p* < 0.0001).

The VWA was maintained at 5.76 ± 2.02° in the LS group and 7.62°±4.43° in the SS group at the 1-year postoperative follow-up; however, there was a statistically significant difference (*p* = 0.047).

The AVBH was maintained at 0.95%±0.02% in the LS group compared to 0.88%±0.02% in the SS group at the 1-year postoperative follow-up; however, there was a statistically significant difference (*p* < 0.0001).

**Fig. 5:**
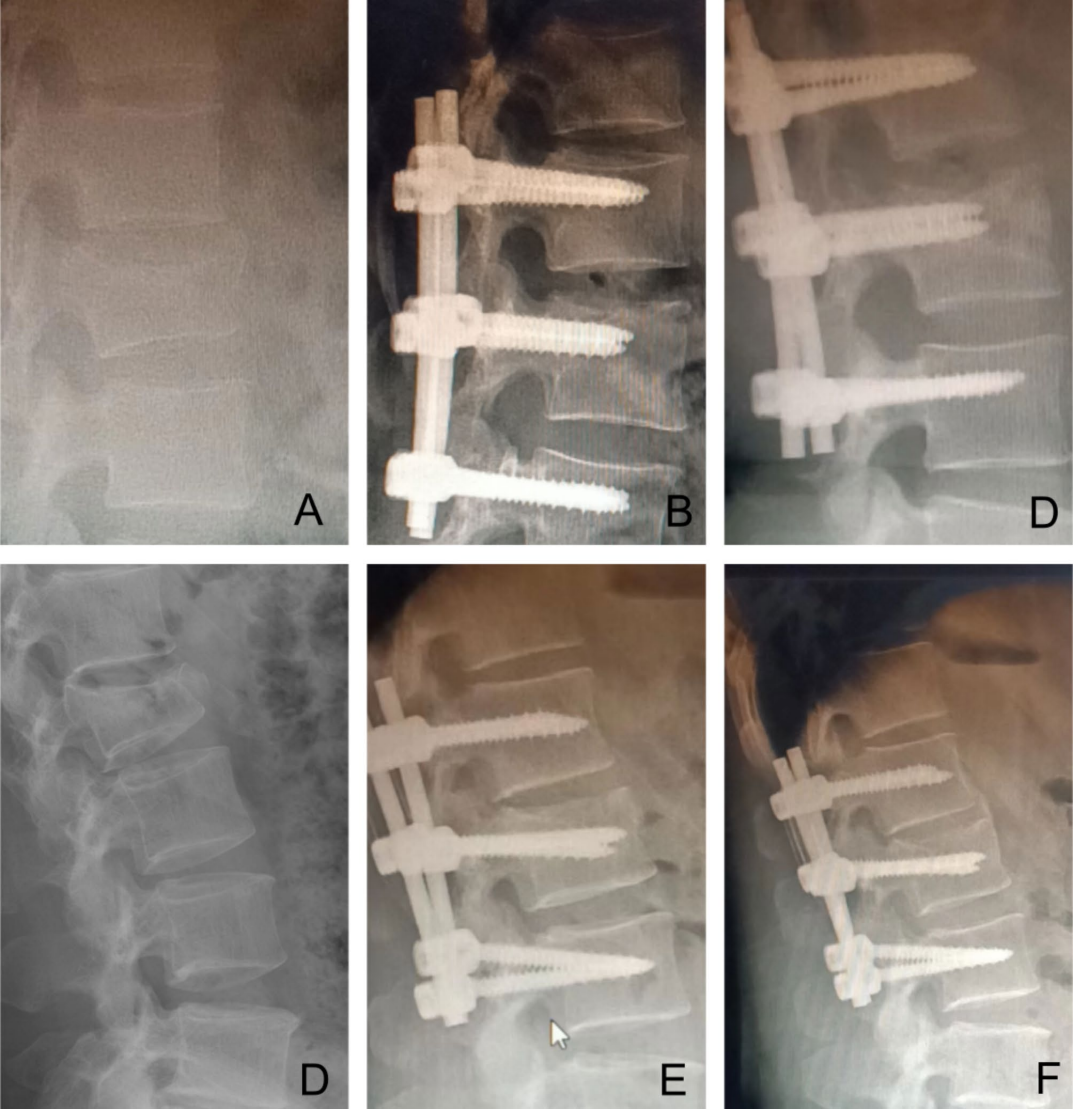
A–C The patient was a 23-year-old male with an L3 fracture sustained by falling from a height (SS group). (**A**): Preoperative lateral radiograph. (**B**) One week after the operation, the vertebra recovered, with little difference from the LS group. (**C**) One year after the operation, the endplate collapsed. **D-F** The patient was a 53-year-old male with and L3 fracture sustained by falling from a height (LS group). (**A**): Preoperative lateral radiograph. (**B**) One week after the operation, the vertebra recovered, with little difference from the SS group. (F) One year after the operation,, the morphology of the vertebral body remained intact

The MVBH was maintained at 0.94%±0.02% in the LS group compared to 0.87%±0.02% in the SS group at the 1-year postoperative follow-up; however, there was a statistically significant difference (*p* < 0.0001) (Table 2; Fig. 5).

**Table 2:** Pre- and postoperative CA, VWA, AVBH, and MVBH

Parameter	LS group (*n* = 54)	SS group (*n* = 39)	*P*	Adjusted *P*
CA (°)				
Preoperative	15.15 ± 5.34	13.4 ± 6.02	0.142^†^	
Postoperative	3.92 ± 0.63	3.95 ± 0.53	0.604^†^	
1-year follow-up	4.36 ± 0.62	6.98 ± 1.42	< 0.0001^‡^	0.039*
Correction loss	0.44 ± 0.3	3.02 ± 1.52	< 0.0001^†^	
VWA (°)				
Preoperative	15.28 ± 5.27	13.43 ± 5.72	0.111^†^	
Postoperative	7.64 ± 3.97	6.83 ± 4.24	0.328^‡^	
1-year follow-up	5.76 ± 2.02	7.62 ± 4.43	0.047^‡^	0.004*
Correction loss	1.88 ± 2.88	-0.79 ± 1	< 0.0001^‡^	
AVBH (%)				
Preoperative	0.62 ± 0.12	0.67 ± 0.14	0.074^†^	
Postoperative	0.96 ± 0.02	0.96 ± 0.02	0.834^†^	
1-year follow-up	0.95 ± 0.02	0.88 ± 0.02	< 0.0001^†^	< 0.0001*
Correction loss	0.01 ± 0.02	0.08 ± 0.02	< 0.0001^‡^	
MVBH (%)				
Preoperative	0.66 ± 0.12	0.67 ± 0.17	0.904^‡^	
Postoperative	0.97 ± 0.02	0.96 ± 0.01	0.641^†^	
1-year follow-up	0.94 ± 0.02	0.87 ± 0.02	< 0.0001^†^	< 0.0001*
Correction loss	0.02 ± 0.01	0.09 ± 0.02	< 0.0001^‡^	

Values are presented as the mean ± standard deviation. LS group, long screw group; SS group, short screw group; VWA, vertebral, wedge angle; CA, Cobb angle; AVBH, anterior vertebral body, height; MVBH, middle vertebral body height; *p < 0.05 was considered statistically significant. Adjusted P, means the *p*-value adjusted by Gender, Age, Fracturelevel, classification of fracture, BMI, BMD.† Chi-square test. ‡ Wilcoxon test.*Logistic regression analyses

The preoperative VAS score was 1.48 ± 0.5 and 1.97 ± 0.71 points in the LS group and the SS group, respectively. The VAS score was significantly lower in both groups after surgery, but there was no significant difference between the preoperative and 1-week postoperative scores. However, at the final follow-up, there was a statistically significant difference in the VAS score between the two groups (*P* = 0.001) (Table 3).

**Table 3:** Change in VAS score from pre- to postoperatively

Parameter	LS group	SS group	*P*
Preoperative	8.54 ± 0.61	8.77 ± 0.67	0.116^‡^
Postoperative	4.65 ± 1.48	4.82 ± 0.72	0.159^‡^
1-year follow-up	1.48 ± 0.5	1.97 ± 0.71	0.001^‡^
Correction loss	3.17 ± 1.51	2.85 ± 0.96	0.321^‡^

Values are presented as the mean ± standard deviation. LS group, long screw group; SS group, short screw group; *p < 0.05 was considered statistically significant. †Chi-square test. ‡ Wilcoxon test

At the 1-year postoperative follow-up, the ODI was maintained at 8.3 ± 1.42 in the LS group compared with 12.41 ± 2.64 in the SS group; however, there was a significant difference (*p* < 0.0001) (Table 4).

**Table 4:** Change in ODI from pre- to postoperatively

Parameter	LS group	SS group	*P*
Preoperative	85.67 ± 2.66	86.82 ± 2.54	0.056^‡^
Postoperative	52.85 ± 4.72	50.92 ± 3.17	0.062^‡^
1-year follow-up	1.48 ± 0.5	1.97 ± 0.71	< 0.0001^‡^
Correction loss	44.56 ± 5.04	38.51 ± 3.72	< 0.0001^‡^

Values are presented as the mean ± standard deviation. LS group, long screw group; SS group, short screw group; *p < 0.05 was considered statistically significant. †Chi-square test. ‡ Wilcoxon test

## Discussion

### Short- or long-segment fixation

There have been many treatments for thoracolumbar fractures. Currently, the most popular treatment is posterior pedicle fixation [19]. Additionally, the majority of treatments for thoracolumbar fractures have consisted of long-segment fixation techniques [2]. Long-segment fixation can provide strong fixation to facilitate early functional exercise while avoiding the risk of implant breakage and posttraumatic kyphosis [11]. However, there is still a great deal of controversy about whether long-segment fixation is necessary. Longer-segment fixation is associated with a longer operation, more bleeding, and a greater chance of infection. Additionally, long-segment fixation of the spine not only increases the load and motion of the immediately adjacent segments but also increases the burden on the intervertebral discs and accelerates their degeneration. In recent years, more studies have shown greater benefits of short-segment fixation for thoracolumbar fractures. Compared to long-segment fixation, short-segment fixation is associated with less blood loss, a shorter operation, and fewer fused segments. Girardo, M. et al. reported that short- and long-segment fixation showed good clinical and radiological results [20]. In this retrospective study, we followed up with all patients who underwent short-segment fixation after one year, and none of them had experienced complications such as implant breakage or severe posttraumatic kyphosis. Therefore, short-segment fixation can provide strong internal fixation.

### Necessity of intermediate screws

Short-segment fixation requires less time, leading to less bleeding and a lower chance of infection. However, studies have shown that this technique also carries risks of adverse events such as implant fracture and posttraumatic kyphosis. Liang, C. et al. [21] confirmed that vertebral body fracture causes compression of the cancellous bone, leaving a void in the vertebral body after endplate relocation. If this cavity is not filled effectively, vertebral bone defects of varying degrees can occur, and this cavity is one of the main risk factors for loss of height of the injured vertebra after fracture correction. If the cavity results in a defect in the anterior weight-bearing column of the spine, when standing, especially during flexion-extension and lateral bending, axial compressive forces the fractured vertebrae can result in loss of vertebral reduction and recurrent kyphosis [22]. Therefore, the early postoperative phase may be effectively supported by the use of intermediate screws, which distribute the stress on the anterior column of the fractured vertebral body and prevents endplate collapse and posterior convex deformity [23]. El Behairy, H.F. et al. [1] showed that fixation using short-segment pedicle screws as well as intermediate screws prevented postoperative vertebral wedge deformation, vertebral body height reduction, and segmental kyphosis over a two-year follow-up period. The results of this research support previous findings in the literature suggesting that short-segment fixation with bilateral intermediate screws is sufficient to rectify kyphosis and provide spinal stability in the treatment of thoracolumbar fractures [5, 24, 25]. According to Farrokhi et al. [23], intermediate screws provide similar clinical results and better kyphosis correction with fewer implant failures. According to the current study, intermediate screws can provide increased support for the fractured segment of the pedicle, improve the stress on the internal fixation system through pedicle screw fixation of the fractured vertebra, and further decrease the likelihood of kyphosis through three-point fixation. Three-point fixation can reduce the suspension effect of the internal fixation system, further reduce the occurrence of retroflexion deformity, and increase the stability of the internal fixation system [24, 26]. In our study, the fractured vertebrae were well repositioned using intermediate screws in both the LS and SS groups, with positive clinical and radiological outcomes, even in cases of fractures with extensive spinal comminution and kyphosis, according to the one-year follow-up data.

### Necessity of longer screws

According to recent research, there is no standard for the depth of intermediate screw insertion. In an attempt to reduce the possibility of screw failure due to loosening, the screw insertion depth has been extensively researched [27–30]. Through biomechanical analysis, Liu, J. et al. showed that the loss of height correction of the fractured vertebra is tolerated when the screw is inserted to 60% of the vertebral body [31]. Another study reported that increasing the screw depth from 50 to 80% increases the stability of the screw under flexural and torsional loads by 30%. In a biomechanical investigation, Matsukawa, K. et al. found that the chance of screw loosening decreased with increasing screw depth [29]. In fact, many scholars have questioned the safety of placing screws in fractured vertebrae, as the benefits must be weighed against the corresponding risk of accelerated deterioration. However, our study showed no further fracture displacement or nerve damage in any patient treated with pedicle screws. This study focused on the clinical and imaging results of thoracolumbar fracture fixation with various screw insertion depths in the damaged vertebrae. Follow-up radiological and clinical evaluations showed satisfactory repositioning of the fractured vertebrae and satisfactory recovery of the anterior vertebral body height and Cobb angle in both groups of patients, with no statistically significant difference between the LS and SS groups. We believe that regardless of screw length, there is little effect on repositioning of the injured spine. However, the results of the one-year follow-up showed 0.01%±0.02% loss of correction in the LS group, with almost no change in the AVBH, compared with 0.08%±0.02% in the SS group (*p* < 0.0001). Additionally, the SS group showed even less effective Cobb angle maintenance; the CA was corrected to 3.02 ± 1.52°. The insertion depth in the LS group on the lateral view reached 60% of the vertebral body length, which offered greater support, according to our retrospective research, indicating that longer screws may better retain the morphology of the anterior column of the spine. To improve the stability of the anterior column of the collapsed vertebral body, we think it is essential to insert a longer screw, which offers several advantages: Longer pedicle screws improve the immediate stability and stiffness of damaged spinal segments, providing effective support to the endplate and preventing endplate collapse and disc protrusion into the vertebral body. Longer pedicle screws also strengthen the anterior spinal column to help prevent kyphosis deformity. Furthermore, longer pedicle screws can act as a prying device to assist in the repositioning of the collapsed anterior spinal column.

This study has some limitations. First, this research was retrospective, and the sample size was relatively small. Second, since the research period was only one year, the findings could not be indicative of the long-term outcomes. Finally, the effect of disc endplate fracture could not be studied further. Further studies of MRI data will be performed to observe and analyze metrics of disc degeneration.

In conclusion, a longer screw provides greater grip on the fractured vertebral body and more support for the vertebral plate. On the lateral view, the ideal screw placement depth surpasses 60% of the vertebral body length.
